# Incidence of and risk factors for nephrolithiasis in patients with gout and the general population, a cohort study

**DOI:** 10.1186/s13075-017-1376-z

**Published:** 2017-07-24

**Authors:** A. J. Landgren, L. T. H. Jacobsson, U. Lindström, T. Z. S. Sandström, P. Drivelegka, L. Björkman, E. Fjellstedt, M. Dehlin

**Affiliations:** 1 Department of Rheumatology and Inflammation Research, Institute of Medicine, Sahlgrenska Academy, University of Gothenburg, Guldhedsgatan 10A, 405 30 Gothenburg, Sweden; 2 Department of Nephrology and Transplantation, SUS University Hospital, Malmö, Sweden

**Keywords:** Epidemiology, Gout, Incidence, Predictors, Nephrolithiasis

## Abstract

**Background:**

Nephrolithiasis (NL) is known to be associated with gout, although there are few comparative studies on risk and risk factors for NL in gout compared to population cohorts. In this cohort study we investigated: (1) overall incidence of NL in gout (cases) and general population controls; (2) risk and risk factors (common comorbidities and medications) for first-time NL in cases and controls separately.

**Methods:**

Cases (*n* = 29,968) and age-matched and sex-matched controls (*n* = 138,678) were identified from the regional healthcare database in western Sweden (VEGA). The analyzed risk factors (comorbidities and current medication use) for first-time NL, and socioeconomic factors were retrieved from VEGA and other national Swedish registers. For cases, follow up began on 1 January 2006 or on the first diagnosis of gout if this occurred later, and for controls on their index patient’s first diagnosis of gout. Follow up ended on death, emigration or 31 December 2012. Incidence rates (IR) per 1000 person-years and hazard ratios (HR) were calculated. The incidence calculations were performed for cases (regardless of prior NL) and their controls. HRs with first occurrence of NL as outcome were calculated only in those without previous NL.

**Results:**

In cases there were 678 NL events (IR: 6.16 events per 1000 person-years (95% CI: 5.70–6.64) and in controls 2125 NL events (IR 3.85 events per 1000 person-years (95% CI: 3.69–4.02), resulting in an age-sex-adjusted incidence rate ratio of 1.60 (95% CI:1.47–1.74).

Point estimates for predictive factors were similar in cases and controls, except for a significant interaction for losartan which increased the risk of NL only in controls (HR = 1.49 (95% CI: 1.03–2.14). Loop diuretics significantly decreased the risk of NL by 30–34% in both cases and controls. Further significant predictors of NL in gout cases were male sex, diabetes and obesity and in controls male sex and kidney disease.

**Conclusions:**

The risk (age and sex adjusted) of NL was increased by 60% in cases compared to controls. None of the commonly used medications increased the risk of NL in gout patients.

**Electronic supplementary material:**

The online version of this article (doi:10.1186/s13075-017-1376-z) contains supplementary material, which is available to authorized users.

## Background

Gout is the most common inflammatory joint disease, with a reported prevalence between 1% and 3.9% [1–3], and recently reported in Western Sweden to be 1.8% [4]. A well-known complication of gout is an increased risk of nephrolithiasis (NL) [5–7]. The incidence rate of NL in the general population varies in different studies between 0.85 and 1.70/1000 person-years [8, 9], with a peak incidence at age 40 − 49 years [8, 10, 11]. In contrast, in a large population-based survey 14% of subjects with gout reported a previous episode of NL, and the age-adjusted risk of NL was doubled in subjects with gout, compared to subjects without gout [5]. The increased occurrence of NL in gout could be explained by specific mechanisms related to gout or hyperuricemia, medications given to patients with gout or shared etiological factors between NL and gout, such as comorbidities and pharmacological treatment.

In particular, NL composed of uric acid is considerably more common in patients with gout [5, 12, 13], which is at least partly explained by increased urine levels of uric acid [13]. Stone formation in patients with gout has been correlated with hyperuricemia, hyperuricosuria and low urinary pH [7]. Apart from causing uric acid NL, hyperuricosuria may also decrease the solubility of calcium oxalate (CaOx), and hyperuricosuria has been proposed as a risk factor for CaOx stones as well in some [14, 15] but not all studies [16, 17]. In addition, several medications used in patients with gout could affect the risk of NL, including allopurinol, which decrease urate production [18].

Several risk factors for NL in general, including older age [19], male sex [9], obesity and hypertension [9], diabetes mellitus (DM) [20] and kidney disease (KD) [21], are also risk factors for developing gout [22]. Several types of medication that are frequently used in these conditions also act by mechanisms that could affect stone formation, including the angiotensin II receptor blocker losartan, which has uricosuric effects [23], thiazide diuretics which in high doses may decrease the risk of calcium-containing NL [18] and loop diuretics [24]. In addition several other frequently used medications, for associated comorbidities in gout, have been suggested to affect the risk of gout, whereas their effect on NL is unclear, including calcium channel blockers [25, 26], beta blockers and aldosterone receptor blockers [25], renin-angiotensin-aldosterone-inhibitors (RAAS)-inhibitors [25] and lipid lowering drugs [27–29].There are few prospective cohort studies that have studied these predictors for NL specifically in patients with gout [30].

There is thus a need for contemporary studies comparing the risk of NL in patients with gout to the general population, and evaluating the effect of possible predictors of NL in patients with gout. In particular, the aforementioned modifiable risk factors need to be investigated. In this cohort-study we investigated: (1) overall incidence of NL in gout (cases) and general population controls; (2) risk and risk factors (common comorbidities and medications) for first-time NL in cases and controls separately.

## Methods

### Study design

We performed a cohort study, with two parallel cohorts, one with gout and one without gout (matched general population (GP) controls), based on linkage of healthcare registers in Sweden. Two study designs were used; first, the overall incidence of individual NL events was calculated, using all patients with gout and all GP controls; second, the risk and predictors of new-onset NL were determined separately in the two cohorts through proportional hazard models, excluding individuals with NL prior to the start of follow up. Ethical approval for the study was granted from the Ethical Review Board of Gothenburg, Sweden.

### Setting and study population

The study population consisted of all inhabitants above 19 years of age in the Western Swedish Health Care Region (WSHCR) from 1 January 2006 to 31 December 2012, with a population that is approximately 20% of the total population of Sweden and is considered to be representative of Sweden as a whole with regard to health status and demographics [31].

### Data sources

The Västra Götaland Health Care Register (VEGA) was used to identify cases of gout and the occurrence of NL and comorbidities in both cases and GP controls (for ICD-10 codes used see Additional file 1: Table S1). Matched GP controls and demographic data were obtained from the Swedish population register (http://www.scb.se/). The Prescribed Drug Register (PDR) (http://www.socialstyrelsen.se/register/halsodataregister/lakemedelsregistret) was used to determine drug exposure for cases and controls, the Anatomical Therapeutic Chemical Classification System (ATC) codes used are presented in Additional file 1: Table S2. The Longitudinal Integration Database For Health Insurance And Labor Market Studies (LISA) was consulted to obtain data on the level of education (http://www.scb.se/en_/Services/Guidance-for-researchers-and-universities/SCB-Data/Longitudinal-integration-database-for-health-insurance-and-labour-market-studies-LISA-by-Swedish-acronym/). The Cause-Of-Death Register was used to determine vital status through December 31 2012 (http://www.socialstyrelsen.se/register/dodsorsaksregistret). The registers used in the study are presented in Additional file 1: Table S3.

### Study populations

#### Gout cohort

The gout cohort consisted of patients above the age of 19 years with an ICD-10-code for gout (M10 or M14) at a visit to a physician. The total cohort was used to determine the overall incidence rates of NL, whereas to determine the risk and predictors for new onset NL, only the subgroup without previous NL during a period of at least 6 years preceding the start of follow up was included.

#### Reference cohort

Up to five matched GP controls, without any registered diagnosis of gout in the VEGA database at the time of the index patient’s first gout diagnosis, were identified for each patient with gout. GP controls were matched on year of birth, sex and municipality at the index date, from the population register held by Statistics Sweden. Prior users of urate-lowering medications were excluded from the control cohort. In addition, GP controls who developed gout during the follow-up period were excluded from the analyses. Overall incidence rates and predictors of NL were calculated in the same manner as for the gout cohort.

#### Exposures

Comorbidities were defined as at least one visit to a physician in primary or specialized care with a corresponding ICD-10 code for hypertension, ischemic heart disease (IHD), DM, KD or obesity. For obesity, the prescription of anti-obesity treatment at any time point prior to the start of follow up was also included in the exposure definition (Additional file 1: Table S1). Diagnoses in Swedish registers have previously been shown to have high validity, with a positive predictive value ranging from 85 to 95% for most chronic diseases [32]. In Sweden, medications are usually prescribed for a period of 3 months. Therefore, drug exposure was defined as having a dispensed prescription, in the PDR within 90 days before the start of follow up for the following groups of drugs: statins, allopurinol, beta blockers, calcium antagonists, thiazide diuretics, losartan, potassium-sparing diuretics, RAAS-inhibitors or loop diuretics. Due to its specific uricosuric effect, losartan was analyzed separately.

#### Follow up and outcome

The start of follow up was 1 January 2006, or later in the case of a first diagnosis of gout occurring after this date for cases. Controls have been assigned their index patient’s start date. This date was chosen in order to enable assessment of baseline data and risk factors from the available registers, and to have a period of at least 6 years free of NL.

Time at risk, both in the incidence calculations and the proportional hazard analyses was from study entry until 31 December 2012, death, emigration or first NL diagnosis during follow up, whichever came first.

The outcome was an episode of NL. For the calculation of incidence this was defined as having been given an ICD-10 code for NL at a visit to a physician, without having received such a code during the preceding 6 months. One individual could thus have several NL events. In the proportional hazard analyses, outcome was defined as the first occurrence of NL after the start of follow up, excluding those with an ICD-10 code of NL in the VEGA-register before the start of follow up.

#### Statistics

Frequencies were computed for the baseline variables. Absolute risks (incidence per 1000 person-years with 95% confidence interval), and the incidence rate ratio were calculated assuming a Poisson distribution. In addition, incidence rates were calculated for individuals without prior NL (Additional file 1: Table S4). The hazard ratios (HR) for cases versus controls were calculated using age-adjusted and sex-adjusted Cox proportional hazard regression analyses, both overall and stratified by sex (Additional file 1: Figure S1 and S2).

**Fig. 1:**
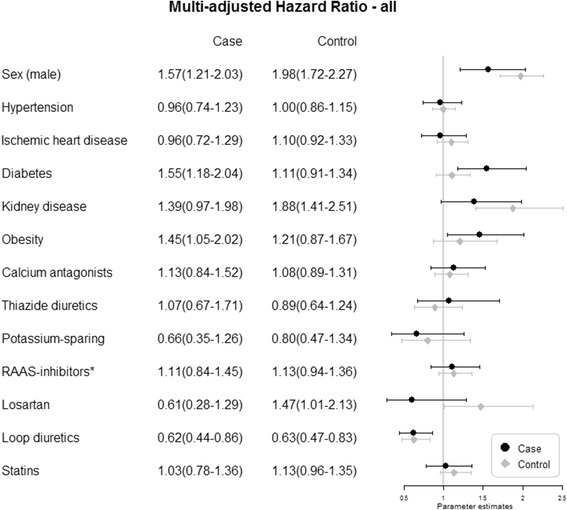
Predictors for first time nephrolithiasis in patients with gout (*Cases*) and general population controls without gout (*Controls*), analyzed by multivariate proportional hazards analyses, adjusting for age and the other covariates in the figure. *Renin-angiotensin-aldosterone-system-inhibitors (*RAAS*) excluding losartan

Predictors for NL were first evaluated using an age-adjusted and sex-adjusted Cox proportional hazard regression model for patients with gout and GP controls separately. The predictors were then entered into multiple Cox regression models (Fig. 1). The possible confounder “level of education” was not entered into the multiple regression model, because the first age-adjusted and sex-adjusted analysis did not indicate a significant effect. Further, allopurinol was also excluded as it was per definition excluded from the GP-control cohort.

Sensitivity analyses were performed to evaluate the possible effect of prolonged exposure to various medications, whereby exposure was defined as having at least one batch of the medication dispensed prior to the start of follow up and an additional batch dispensed during follow up. Non-exposure in comparison analyses was defined as having no medication dispensed prior to the start of follow up and no medication dispensed during follow up.

Bivariate correlation was tested between all co-variates included, to assess collinearity in the multiple regression model. In bivariate correlation analyses an absolute value of the Spearman coefficient higher than or equal to 0.4 was considered to indicate collinearity. Of the covariates only hypertension and use of beta blockers in controls fulfilled this definition. Beta blockers were excluded from the final multiple regression analysis as a consequence of this.

Interactions between medical exposures that predicted NL in cases or controls (current losartan and loop-diuretic exposure) and other covariates were systematically sought. To explore if exposure differed significantly between cases and controls, interactions between case-control status and all the analyzed exposures were systemically sought in analyses of cases and controls combined, by creating interaction variables between case-control status and exposure status, in age-adjusted and sex-adjusted Cox regression models. All analyses were conducted using SAS software, version 9.3 (SAS institute Inc. Cary, NC, USA). The significance level was set at alpha <0.05.

## Results

### Incidence rates and relative risks for individual and first time NL events

In total, 29,968 patients with gout and 138,678 matched GP controls were included. The overall incidence rate for individual NL events was 6.2 (95% CI: 5.7–6.6) and 3.9 (95% CI: 3.7–4.0) per 1000 person-years in patients with gout and GP controls, respectively (Table 1). The incidence rate ratio between cases and controls was 1.60 (95% CI: 1.47–1.74). In cases, the highest incidence rate was seen in men 20–39 years old. In controls, the highest incidence rate was seen in the group of men 60–79 years old.

**Table 1:** Incidence rates (including all individual nephrolithiasis (NL) events without exclusion of subjects with NL before start of follow up) for NL in patients with gout and general population (GP) controls by sex and age groups and overall, with 95% confidence interval

	Gout cases (*n* = 29,968)			GP controls (*n* = 138,678)		
	NL events, *n*	Person-years at risk, *n*	Incidence rate per 1000 person-years (95% CI)	NL events, *n*	Person-years at risk, *n*	Incidence rate per 1000 person-years (95% CI)
Women						
20‒39 years	8	1130	7.2 (3.2‒14.0)	18	5487	3.3 (1.9‒5.2)
40‒59 years	44	6363	6.9 (5.0‒9.3)	64	32,015	2.0 (1.5‒2.6)
60‒79 years	74	17,043	4.3 (3.4‒5.5)	212	87,677	2.4 (2.1‒2.8)
80+ years	35	9598	3.7 (2.5‒5.1)	71	51,216	1.4 (1.1‒1.8)
Men						
20‒39 years	35	4571	7.7 (5.3‒10.7)	68	22,323	3.1 (2.4‒3.9)
40‒59 years	177	23,267	7.6 (6.5‒8.8)	552	115,562	4.8 (4.4‒5.2)
60‒79 years	266	37,896	7.0 (6.2‒7.9)	966	187,501	5.2 (4.8‒5.5)
80+ years	39	10,205	3.8 (2.7‒5.2)	174	49,622	3.5 (3.0‒4.1)
Women	161	34,134	4.7 (4.0‒5.5)	365	176,395	2.2 (1.9‒2.3)
Men	517	75,939	6.8 (6.2‒7.4)	1760	375,008	4.7 (4.5‒4.9)
Total	678	110,073	6.2 (5.7‒6.6)	2125	551,403	3.9 (3.7‒4.0)

The risk of first time NL during the follow-up period was overall higher in patients with gout compared to controls (age-adjusted and sex-adjusted HR = 1.49, 95% CI: 1.35–1.64) and were higher in men compared to women (age-adjusted) both in patients with gout (HR = 1.21, 95% CI: 1.09–1.35) and in GP controls (HR = 1.46, 95% CI: 1.37–1.55).

In the population without prior NL, including 29,171 patients with gout and 131,449 controls, the incidence rates for individual NL events (Additional file 1: Table S4) were overall slightly lower.

### Comorbidities and medications at baseline

In the analyses of predictors, only subjects without prior history of NL before the start of follow up were included. All comorbidities and medications (Table 2) were significantly more frequent in gout cases compared to controls (*p* values <0.0001 for all variables) at baseline, and known predictors of NL such as KD, obesity and DM were two to four times more common in the patient with gout. The relative differences between the point estimates in patients with gout and GP controls were similar in men and women (Additional file 1: Table S5).

**Table 2:** Baseline characteristics in patients and GP controls without a previous history of NL, given as frequencies (%)

Comorbidities and medications at baseline	Gout cases (*N* = 29,171)	GP controls (*N* = 131,449)
Age, mean (std)	69.1 (14.8)	68.2 (14.6)
Men (%)	67.3	66.3
Hypertension (%)	58.4	33.0
Ischemic heart disease (%)	26.6	13.5
Diabetes (%)	18.6	9.2
Kidney disease (%)	11.1	2.6
Obesity^a^ (%)	9.9	3.1
Statins (%)	32.0	19.3
Allopurinol (%)	26.4	N/A^c^
Beta blockers (%)	38.4	20.1
Calcium antagonists (%)	16.3	10.9
Thiazide diuretics (%)	6.1	3.6
Losartan (%)	4.0	2.0
Potassium-sparing diuretics (%)	6.7	2.1
RAAS-inhibitors (%)	24.9	12.2
Loop diuretics (%)	27.9	7.9
Education (≤9) years (ref)^b^ (%)	44.7	41.0
Education (10‒12) years^b^ (%)	37.8	36.9
Education (>12) years^b^ (%)	15.5	20.3

*NL*, nephrolithiasis, *RAAS* renin-angiotensin-aldosterone-system, *N/A* not applicable

^a^Based on ICD-10-code E66 and ATC code A08

^b^Baseline data were complete except for data on education level, which was missing for 1.8% of the GP controls and 2 percent of the gout cases.

^c^Prior users of urate-lowering-therapy were excluded from the control group

### Predictors of first-time NL in cases and controls

Overall the point estimates for comorbidities and medications followed similar directions in patients with gout and GP controls in both the age-adjusted and sex-adjusted proportional hazards models (Table 3), with the exception of losartan. In the age-adjusted and sex-adjusted proportional hazards models, DM and obesity significantly increased, and medication with loop diuretics decreased, the risk of first-time NL in patients with gout. In controls, ischemic heart disease, KD and medication with losartan or statins significantly increased, and medication with loop diuretics decreased, the risk of first-time NL. Allopurinol did not predict NL in patient with gout. However, the doses of allopurinol used were low, with 62% of patients prescribed 100 mg per day.

**Table 3:** Predictors of first-time NL in patients with gout and GP controls, analyzed by age- and sex-adjusted proportional hazards analyses

Variable	Gout cases	GP controls
	(*N* = 29,171)	(*N* = 131,449)
	(95% HR)	(95% HR)
Male sex	1.56 (1.21‒2.02)^b^	1.98 (1.72‒2.27)^b^
Hypertension	1.11 (0.89‒1.38)	1.11 (0.98‒1.25)
Ischemic heart disease	0.97 (0.74‒1.27)	1.18 (1.00‒1.38)
Diabetes	1.57 (1.21‒2.02)	1.19 (0.99‒1.43)
Kidney disease	1.32 (0.94‒1.86)	1.87 (1.40‒2.49)
Obesity	1.55 (1.13‒2.12)	1.27 (0.92‒1.75)
Calcium antagonists	1.18 (0.89‒1.56)	1.14 (0.96‒1.36)
Thiazide diuretics	1.21 (0.77‒1.90)	1.00 (0.72‒1.38)
Potassium-sparing diuretics	0.58 (0.31‒1.10)	0.74 (0.44‒1.23)
RAAS-inhibitors^a^	1.13 (0.89‒1.45)	1.15 (0.97‒1.35)
Losartan	0.61 (0.29‒1.29)	1.49 (1.03‒2.14)
Loop diuretics	0.71 (0.52‒0.96)	0.73 (0.56‒0.95)
Statins	1.06 (0.84‒1.33)	1.28 (1.12‒1.47)
Beta blockers	0.86 (0.68‒1.09)	1.07 (0.93‒1.23)
Allopurinol	1.01 (0.80‒1.28)	N/A^c^
Education (≤9) years (ref)	Ref	Ref
Education (10‒12) years	1.06 (0.84‒1.33)	0.99 (0.87‒1.12)
Education (>12) years	0.98 (0.72‒1.34)	0.92 (0.79‒1.07)
Age 20‒29 years (ref)	Ref	Ref
Age 30‒39 years	1.06 (0.37‒2.99)^d^	0.95 (0.49‒1.83)^d^
Age 40‒49 years	1.19 (0.37‒3.80)^d^	1.34 (0.67‒2.67)^d^
Age 50‒59 years	1.42 (0.35‒5.74)^d^	1.44 (0.65‒3.19)^d^
Age 60‒69 years	1.47 (0.28‒7.86)^d^	1.80 (0.72‒4.55)^d^
Age 70‒79 years	1.48 (0.20‒10.82)^d^	1.71 (0.58‒5.01)^d^
Age 80‒89 years	1.35 (0.14‒13.37)^d^	1.33 (0.39‒4.54)^d^
Age 90‒99 years	0.37 (0.02‒9.46)^d^	1.22 (0.28‒5.28)^d^

*NL*, nephrolithiasis, *GP* general population, *HR* hazard ratio, *RAAS* renin-angiotensin-aldosterone-system, *N/A* not applicable

^a^Excluding losartan

^b^Age-adjusted

^c^Prior users of urate-lowering therapy were excluded from the control group

^d^Sex-adjusted

In the multivariate models (Fig. 1) adjusted for age, sex and other covariates considered as possible risk factors, directions and magnitudes of point estimates were overall similar to those in the models adjusted for age and sex. Losartan predicted NL only in GP controls, with a non-significant protective effect in patients with gout. Regarding comorbidities, DM and obesity significantly predicted NL in patients with gout. Furthermore, KD significantly predicted NL in GP controls.

Regarding medication, losartan significantly predicted NL in GP controls (HR = 1.47, 95% CI: 1.01–2.13) but not in patients with gout (HR = 0.61, 95% CI: 0.28–1.29) and loop diuretics decreased the risk for NL in both patients with gout and GP controls. Medication with thiazide diuretics, calcium channel blockers, statins, potassium-sparing diuretics or RAAS-inhibitors did not significantly affect the risk of NL in the multivariate analyses.

### Additional analyses

First, analyses were stratified by sex (Additional file 1: Figures S1 and S2), which resulted in similar point estimates for risk factors, but with wider confidence intervals.

Second, exploration of possible interactions of losartan and loop diuretics with other possible predictors of NL, showed a significant interaction between loop diuretics and hypertension, (*p* = 0.007) in controls, and between losartan and RAAS inhibitors excluding losartan (*p* = 0.023) in cases. The point estimate HR for losartan in cases was unchanged when adjusting for this interaction. The protective effect of loop diuretics in controls was no longer statistically significant when adjusting for such interaction between hypertension and loop diuretics, indicating that use of loop diuretics may only be protective in subjects with a diagnosis of hypertension.

Third, to explore if predictors differed between cases and controls significant interactions were systematically sought. The only significant interaction was between losartan and having gout (*p* = 0.036).

Fourth, in order to explore whether prolonged exposure to various medications compared to no exposure during follow up changed the risk estimates, sensitivity analysis was performed for the exposure to medications. In these age-adjusted and sex-adjusted analyses (Additional file 1: Table S6), exposure was defined as having at least one batch of the medication dispensed prior to the start of follow up and an additional batch of the medication dispensed during follow up. Non-exposure was defined as having no medication dispensed prior to the start of follow up and no medication dispensed during follow up. The HR did not change substantially (except for losartan, which in these analyses was associated with a non-significant increased risk of NL in controls). The protective effect of loop diuretics remained significantly protective in both cases and controls.

## Discussion

The incidence of NL was consistently higher in patients with gout in all age and sex groups, compared to GP controls, with the highest incidence in patients with gout ages 20–39 years and in GP controls ages 60–79 years. Further, the risk of first-time NL was increased in patients with gout compared to controls by 60%, with overall similar risk factors, with the exception of losartan exposure, which increased the risk of NL only in GP controls.

Gout has been linked with NL in previous studies [5–7]. A recent meta-analysis reported an overall HR of 1.77 [30], and in another recent analysis of a UK GP cohort an adjusted HR of 1.26 [33] was reported. Our incidence rate ratio of 1.60 is between these two estimates, and the modest differences could well be explained by differences in patient selection. Previously suggested risk factors for NL in general, including older age [19], male sex [9], obesity and hypertension [9], DM [20] and KD [21] were also confirmed in our study, albeit with HR estimates that differed slightly between cases and controls.

Adequately dosed allopurinol treatment decreases the proportion of urate-containing NL in patients with gout [34]. In addition, allopurinol has been shown to have a possible protective effect against recurrent calcium NL in individuals forming calcium stones in one RCT [18, 35]. Possible explanations for why we did not observe a protective effect of allopurinol, could partly be nonadherence and low dosing of urate-lowering therapy (ULT) in clinical practice, problems that we have previously demonstrated in this study population [36]. Suboptimal treatment of gout has previously been shown in a Swedish setting by us [4] and in an international context by others [1, 37, 38]. Had the dosing of allopurinol been optimized, aiming at treatment goals for serum uric acid levels, it is possible that the incidence of NL would have been lower in patients with gout.

Losartan has not been reported to be associated with an increased risk of NL in clinical trials [23]. However, losartan lowers serum uric acid and raises urinary concentration of uric acid with a simultaneously increased excretion of bicarbonate. The latter may counterbalance the effects of uricosuria on NL formation, although both effects have been suggested to wane with exposure time. The net effect of these mechanisms over time is thus difficult to predict. Our results suggest an increased risk of NL in individuals without gout who are treated with losartan. On the other hand it is difficult to draw firm conclusions from such findings, since they may as in all observational studies be affected by residual confounding.

Thiazide diuretics in high doses (50 mg or more hydrochlorthiazide per day) have in a number of RCTs been shown to reduce the recurrence rate of calcium-containing NL [39]. The most likely explanation for why we did not observe a protective effect of such medication, is that the vast majority of patients (>99%) were given low doses (25 mg hydrochlorthiazide per day) for indications other than protection against NL.

Loop diuretics and the risk of NL have, to our knowledge, not been investigated in an adult population. Loop diuretics inhibit sodium and calcium reabsorption in the thick ascending loop of Henle [24] and could theoretically increase the risk of calcium-containing NL. On the other hand loop diuretics have been suggested to increase clearance of NL fragments after extra-corporeal shock-wave lithotripsy [40]. In our study, loop diuretics decreased the risk of NL in both patients with gout and GP controls.

Some limitations in our study should be acknowledged. First, there may have been some misclassification with regard to classification of gout and NL. Our previous validation supports acceptable validity of gout diagnoses in primary care [41]. Second, the definition of NL as an outcome is also inherently difficult due to the episodic recurrent nature of NL. Third, since we only had data covering a minimum of 6 years free from NL prior to the start of follow up, we cannot exclude an infrequent occurrence of NL in the subsample that was used for prediction. Such infrequent occurrence of NL is, however, unlikely to affect medication at baseline and subsequently our risk estimates. Fourth, as in all observational studies there may be a problem with residual confounding. A number of proposed mechanisms for the increased risk of NL in persons with gout include hyperuricemia, high urinary excretion of uric acid, and low urine pH [7], factors that could not be included and assessed in our register-based study. Fifth, some comorbidities that may increase the risk of NL could not be assessed, such as inflammatory bowel disease [42] and primary hyperparathyroidism [43, 44]. In addition, we did not have access to information on previously suggested risk factors for NL, such as diet and body mass index [12].

There are also some strengths to our study. First, it was population-based, which minimizes selection bias. Second, we used several independent sources when defining possible predictors of NL, which increases validity. Third, this is one of the few recent studies describing both the magnitude and risk factors of NL in patients with gout and population controls, and many of the variables examined in our analysis have not, to our knowledge, been examined previously.

## Conclusions

We found the risk of NL to be increased in both male and female patients with gout. The overall pattern of predictors was similar in patients with gout and in population controls. Obesity and use of loop diuretics were identified as the only potentially modifiable risk factors for NL in gout, although increased use of the latter is associated with risk of exacerbation of gout. In this study, none of the other commonly used drugs for cardiovascular disease and hypertension increased the risk of NL in patients with gout, and neither was there a protective effect of allopurinol given in low doses in clinical practice.

## Additional file

Additional file 1: Tables S1-S6. and supplementary **Figures S1-S2.** **Table S1** Definitions of comorbidities by ICD-10-codes given at a visit to physician, based on data in the National Patient Register and dispensed medication in the national Prescribed Drugs Register (ever before start of follow up). **Table S2.** Definitions of co-medications by the Anatomical Therapeutic Chemical (ATC) Classification System for dispensed medication in the national Prescribed Drugs Register. **Table S3.** Description of regional and national registers used in the study. **Table S4.** Incidence rates of NL in cases/controls without prior NL. **Table S5.** Comorbidities and medications at baseline for cases and controls without prior NL, stratified by sex. **Table S6.** Age-adjusted and sex adjusted hazard ratios for first-time NL in cases and controls separately, with exposure defined as having at least one batch of the medication dispensed before start of follow up and at least an additional batch of the medication dispensed during follow up. Non-exposure was defined as having no medication dispensed before follow up and no medication dispensed during follow up. **Figure S1.** Forest plot with hazard ratios for first-time NL in men, in cases with gout and controls separately. **Figure S2.** Forest plot with hazard ratios for first-time NL in women, in cases with gout and controls separately. (DOC 1465 kb)

## Abbreviations

ATC: Anatomical therapeutic chemical classification system
CIs: Confidence intervals
CVD: Cardiovascular disease
DM: Diabetes mellitus
eGFR: Estimated glomerular filtration rate
GP: General population
HRs: Hazard ratios
ICD: International Statistical Classification of Diseases
KD: Kidney disease
NL: Nephrolithiasis
PDR: Prescribed Drug Register
RAAS: Renin-angiotensin-aldosterone system
ULT: Urate-lowering therapy
VEGA: Western Swedish Health Care Register
WSHCR: Western Swedish Health Care Region
