# Supplementary material for: Incidence of and risk factors for nephrolithiasis in patients with gout and the general population, a cohort study
Source: Arthritis Res Ther. 2017 Jul 24;19:173. doi: 10.1186/s13075-017-1376-z (PMC5525359; doi:10.1186/s13075-017-1376-z)
Supplement: Additional file 1: Tables S1-S6. — and supplementary Figures S1-S2. Table S1 Definitions of comorbidities by ICD-10-codes given at a visit to physician, based on data in the National Patient Register and dispensed medication in the national Prescribed Drugs Register (ever before start of follow up). Table S2. Definitions of co-medications by the Anatomical Therapeutic Chemical (ATC) Classification System for dispensed medication in the national Prescribed Drugs Register. Table S3. Description of regional and national registers used in the study. Table S4. Incidence rates of NL in cases/controls without prior NL. Table S5. Comorbidities and medications at baseline for cases and controls without prior NL, stratified by sex. Table S6. Age-adjusted and sex adjusted hazard ratios for first-time NL in cases and controls separately, with exposure defined as having at least one batch of the medication dispensed before start of follow up and at least an additional batch of the medication dispensed during follow up. Non-exposure was defined as having no medication dispensed before follow up and no medication dispensed during follow up. Figure S1. Forest plot with hazard ratios for first-time NL in men, in cases with gout and controls separately. Figure S2. Forest plot with hazard ratios for first-time NL in women, in cases with gout and controls separately. (DOC 1465 kb) [file 13075_2017_1376_MOESM1_ESM.doc]

**Additional file 1**

| **Table S1 Definitions of comorbidities by ICD-10-codes given at a visit to physician, based on data in the National Patient Register and dispensed medication in the national Prescribed Drugs Register (ever before start of follow-up)** | | |
| --- | --- | --- |
| **Comorbidity** | **ICD-10 code** | **ATC code** |
| Gout | M10, M14 |  |
| Hypertension | I10-I15 |  |
| Ischemic heart disease | I20-I25 |  |
| Diabetes | E10-14, O24 |  |
| Kidney disease | N00-N08, N11-22 (excluding N20) |  |
| Obesity/treatment for obesity | E66 | A08 |
| Nephrolithiasis | N20 |  |
| Corresponding ICD-10 codes for comorbidities included in the study. For obesity, the ATC-code A08 (treatment for obesity), was also included. | | |

**Table S2**

**Definitions of comedications by ATC (Anatomical Therapeutic Chemical) Classification System for dispensed medication in the national Prescribed Drugs Register**

| **Medications** | **ATC-codes** | **Exposure time prior to start of follow-up** |
| --- | --- | --- |
| Statins | C10AA | 90 days |
| Allopurinol | M04AA01 | 90 days |
| Beta blockers | C07 | 90 days |
| Calcium antagonists | C08 | 90 days |
| Thiazide diuretics | C03AA | 90 days |
| Treatment for obesity | A08 | Ever |
| Losartan | C09CA01 | 90 days |
| Potassium-sparing diuretics | C03D | 90 days |
| RAAS-inhibitors* | C09AA+ C09CA-C09CA01 | 90 days |
| Loop diuretics | C03C | 90 days |

***Renin-Angiotensin-Aldosterone-System-Inhibitors. Excluding losartan**

The exposure time of 90 days is defined as having a dispensed prescription within 90 days before start of follow-up.

**Table S3**

**Description of regional and national r**egisters used in the study

| **Register** | **Description** | **Use in study** |
| --- | --- | --- |
| VEGA (Vastra Gotaland Health Care Register) | Containing ICD-10 codes from primary and secondary care with start from the year 2000. The register contains the date of contact and both primary and auxiliary diagnoses given by the treating physician. | ICD-10 codes for case identification and comorbidities in primary and secondary care |
| Swedish Prescribed Drug Register | Containing information about all prescribed drugs dispensed by Swedish pharmacies since July 2005 | ATC-codes and prescription dates for all co-medications used in the study |
| Statistics Sweden | Holds data on immigration, emigration and residency as well as data on socio-economic factors (e.g. marital status and level of formal education) for all persons residing in Sweden. | Demographic data such as education and age. |
| Cause of  death register | Containing information regarding date and causes of death for those residing in Sweden, since 1961. | Vital status for cases and controls |
| LISA (Longitudinal integration data base for health insurance and  labour market studies) | The database integrates existing data from the labour market,  educational and social sectors and is updated each year | Data regarding educational level |

**Table S4 Incidence rates of NL in cases/controls without prior NL**

|  | **Cases**  (n=29171) | | | **Controls**  (n=131449) | | |
| --- | --- | --- | --- | --- | --- | --- |
| **Age/sex-groups** |  | **Person-years** | **Incidence rate** |  | **Person-years** | **Incidence rate** |
|  | **NL events** | **at risk** | **per 1000** | **NL** | **at risk** | **per 1000** |
|  | **(n)** |  | **person-years** | **events** |  | **person-years** |
|  |  |  |  | **(n)** |  |  |
| **Women** | 87 | 33648 | 2.6 (2.17-3.2) | 293 | 171300 | 1.7 (1.5-1.9) |
| **20-39** | 8 | 1105 | 7.2 (3.1-14.3) | 15 | 5338 | 2.8 (1.6-4.6) |
| **40-59** | 17 | 6188 | 2.8 (1.6-4.4) | 52 | 30674 | 1.7 (1.3-2.2) |
| **60-79** | 43 | 16833 | 2.6 (1.9-3.4) | 168 | 85374 | 2.0 (1.7-2.3) |
| **80+** | 19 | 9522 | 2.0 (1.2-3.1) | 58 | 49914 | 1.2 (0.9-1.5) |
| **Men** | 352 | 74035 | 4.8 (4.3-5.3) | 1298 | 355580 | 3.7 (3.5-3.9) |
| **20-39** | 29 | 4491 | 6.5 (4.3-9.3) | 54 | 21693 | 2.5 (1.9-3.3) |
| **40-59** | 126 | 22761 | 5.5 (4.6-6.6) | 402 | 110543 | 3.6 (3.3-4.0) |
| **60-79** | 169 | 36789 | 4.6 (3.9-5.3) | 708 | 176361 | 4.0 (3.7-4.3) |
| **80+** | 28 | 9994 | 2.8 (1.9-4.1) | 134 | 46983 | 2.9 (2.4-3.4) |
| **Total** | 439 | 107683 | 4.1 (3.7-4.5) | 1591 | 526880 | 3.0 (2.9-3.2) |

**Table S5**

**Comorbidities and medications at baseline for cases and controls without prior NL stratified by sex**

|  | **Male cases** | **Male controls** | **Female cases** | **Female controls** |
| --- | --- | --- | --- | --- |
| **Comorbidities** | prevalence (%) | prevalence (%) | prevalence (%) | prevalence (%) |
| Hypertension | 54.6 | 29.4 | 66.2 | 40.2 |
| Ischemic heart disease | 26.4 | 14.1 | 27.1 | 12.5 |
| Diabetes | 16.9 | 9.5 | 22.0 | 8.6 |
| Kidney disease | 10.8 | 2.7 | 11.6 | 2.3 |
| Obesity | 8.1 | 2.4 | 11.0 | 3.2 |
| Statins | 32.5 | 19.5 | 31.0 | 18.9 |
| Allopurinol | 26.9 | N/A † | 27.6 | N/A † |
| Beta blockers | 36.1 | 18.3 | 43.3 | 23.7 |
| Calcium antagonists | 16.1 | 10.1 | 16.7 | 12.6 |
| Thiazide diuretics | 5.6 | 2.8 | 7.1 | 5.2 |
| Losartan | 3.7 | 1.8 | 4.6 | 2.3 |
| Potassium-sparing diuretics | 5.3 | 1.6 | 9.6 | 3.1 |
| RAAS inhibitors* | 25.0 | 12.4 | 24.8 | 12.0 |
| Loop diuretics (%) | 23.1 | 6.2 | 37.9 | 11.2 |
| Education (≤9) years | 40.8 | 38.1 | 52.7 | 46.8 |
| Education (10-12) years | 40.4 | 38.9 | 32.6 | 33.1 |
| Education (>12) years | 17.2 | 21.6 | 12.0 | 17.7 |

N/A=Not applicable

***Renin-Angiotensin-Aldosterone-System-Inhibitors. Excluding losartan**

† Prior users of ULT were excluded from control group

**Table S6**

**Age- and sex adjusted hazard ratios for first time NL in cases and controls separately, with exposure defined as having at least one dispension of the medication before start of follow-up and at least an additional dispension of the medication during follow-up. Non-exposure was defined as having no dispension of the medication before follow-up and no dispension of the medication during follow-up.**

| **Variable** | **Gout cases** | **GP controls** |
| --- | --- | --- |
|  |  |
| **Calcium antagonists** | 1.17 (0.88 – 1.56), n=4443 | 1.09 (0.91 – 1.31), n=13701 |
| **Thiazide diuretics** | 1.25 (0.75 – 2.07), n=1334 | 1.00 (0.71 – 1.39), n=4340 |
| **Potassium-sparing diuretics** | 0.58 (0.30 – 1.13), n=1724 | 0.63 (0.36 – 1.12), n=2535 |
| **RAAS-inhibitors*** | 1.14 (0.89 – 1.47), n=6779 | 1.07 (0.90 – 1.27), n=15176 |
| **Losartan** | 0.56 (0.25 – 1.26), n=1054 | 1.32 (0.89 – 1.95), n=2390 |
| **Loop diuretics** | 0.67 (0.49 – 0.93), n=7733 | 0.69 (0.52 – 0.92), n=9830 |
| **Statins** | 1.11 (0.86 – 1.44), n=6113 | 1.12 (0.96– 1.32), n=16495 |
| **Beta blockers** | 0.88 (0.69 – 1.12), n=10732 | 1.00 (0.86 – 1.15), n=25416 |
| **Allopurinol** | 1.01 (0.79 – 1.28), n=7194 | NA |

***Renin-Angiotensin-Aldosterone-System-Inhibitors.**

**Excluding losartan**

**Figure S1. Forest plot with Hazard ratios for first time NL for men, in cases with gout and controls separately.** *


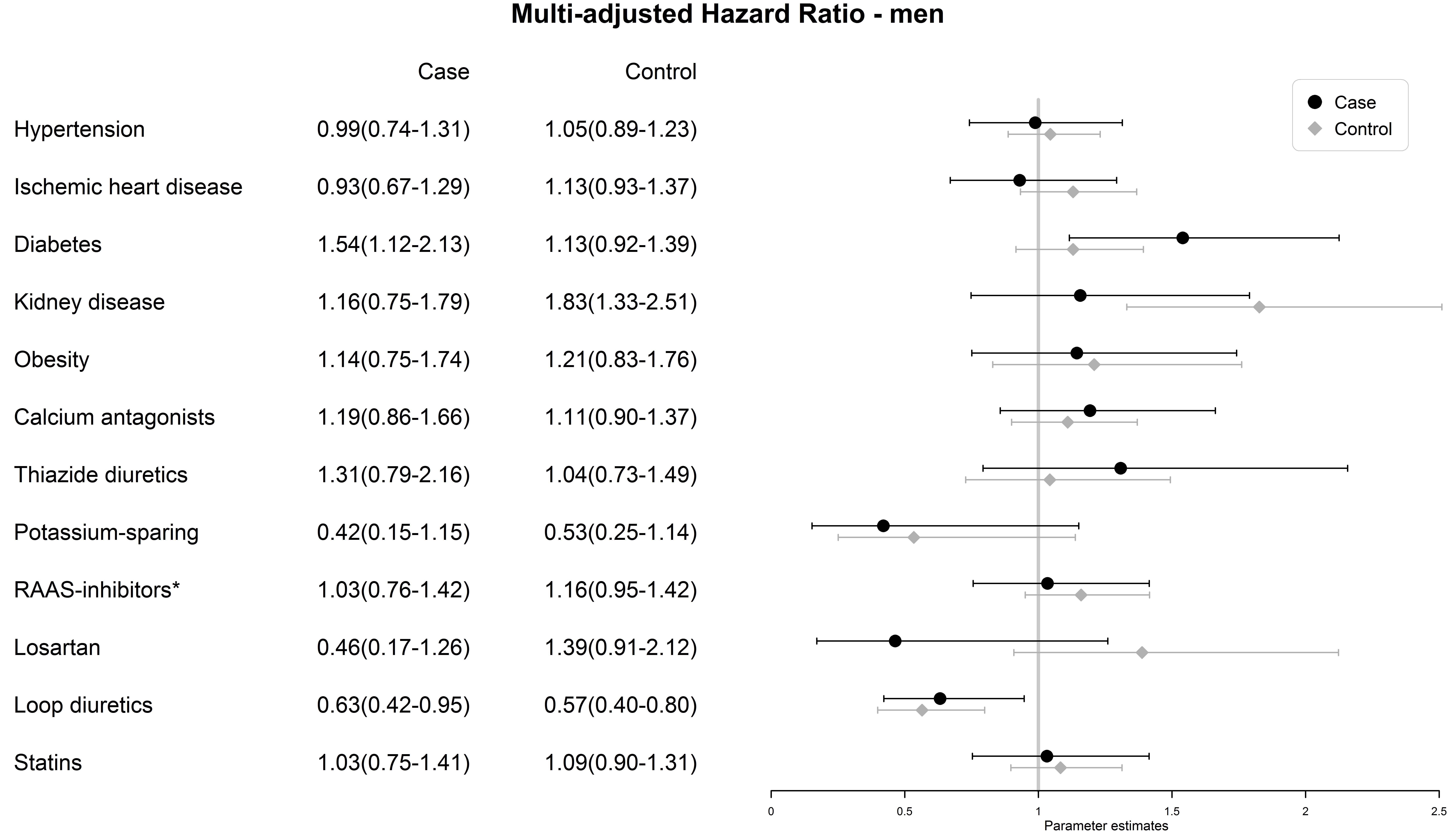


***Renin-Angiotensin-Aldosterone-System-inhibitors** excluding losartan

Hazard ratios for first time NL in gout cohort (n=29171) and control population (n=131449).

**Figure S2. Forest plot with hazard ratios for first time NL in women, in cases with gout and Controls separately**

**
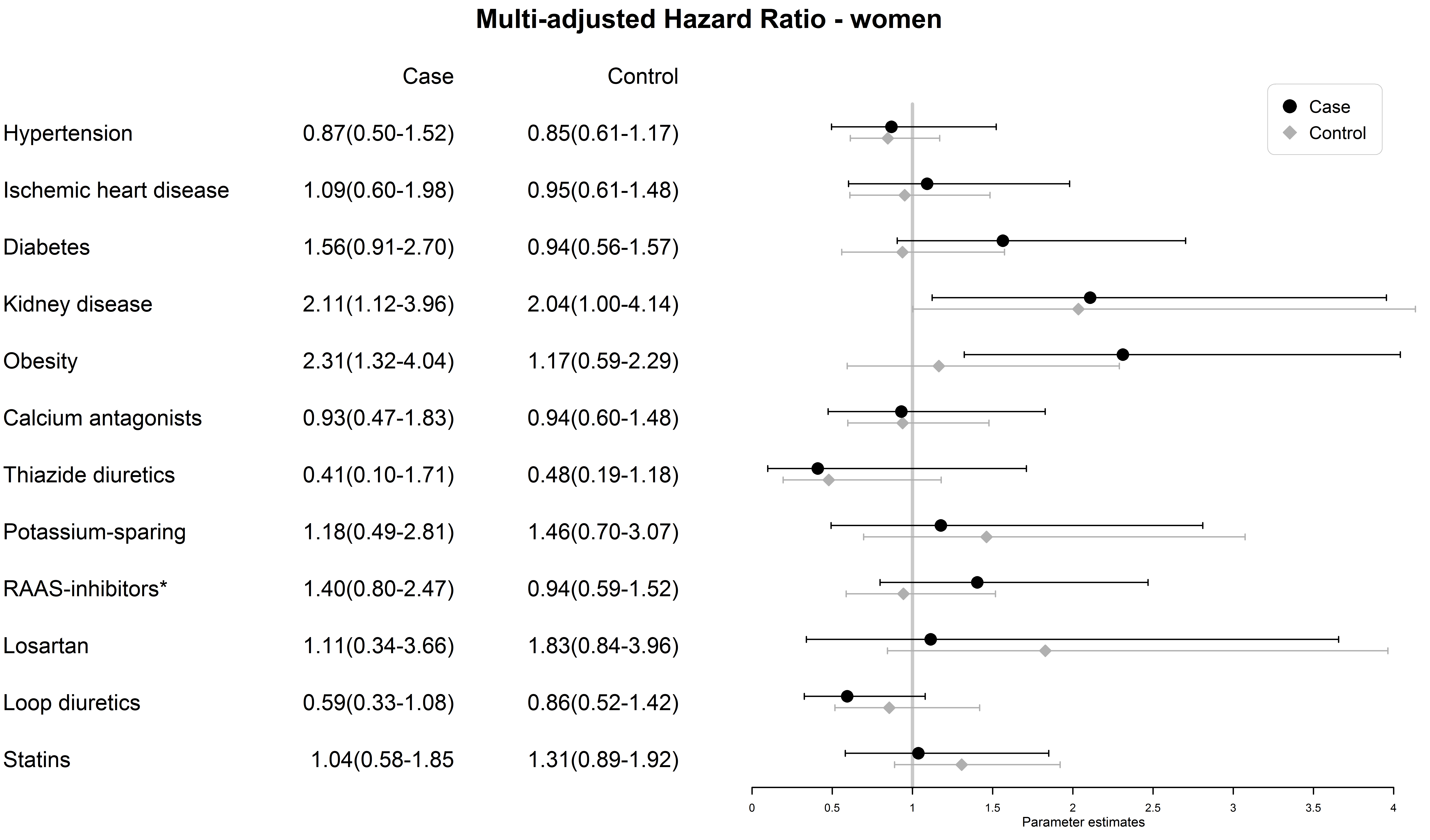
**

***Renin-Angiotensin-Aldosterone-System-inhibitors** excluding losartan

Hazard ratios for first time NL in gout cohort (n=29171) and control population (n=131449).
